# Training load comparison between small, medium, and large-sided games in professional football

**DOI:** 10.3389/fspor.2023.1165242

**Published:** 2023-05-05

**Authors:** Marco Beato, Jordi Vicens-Bordas, Javier Peña, Andrew J. Costin

**Affiliations:** ^1^ School of Health and Sports Sciences, University of Suffolk, Ipswich, United Kingdom; ^2^ Sport and Physical Activity Studies Centre, University of Vic-Central University of Catalonia, Barcelona, Spain; ^3^ Sport, Exercise, and Human Movement, University of Vic-Central University of Catalonia, Barcelona, Spain; ^4^ Department of Sport Science, Ipswich Town FC, Ipswich, United Kingdom

**Keywords:** soccer, team sports, performance, GPS, monitoring

## Abstract

This study aimed to assess if internal and external load parameters were different between sided game formats, if players' positions influenced these parameters, and if load parameters were different among sided game types (from 2vs2 to 10vs10) in professional football players. Twenty-five male players of the same club were enrolled in this study (age = 27 ± 9 years and body mass = 78 ± 14 kg). Sided games were categorized in formats as small-sided games (SSG, *n* = 145), medium-sided games (MSG, *n* = 431), and large-sided games (LSG, *n* = 204). Players were divided into roles such as center backs (CB), fullbacks (FB), center midfielders (CM), attacking midfielders (AM), and strikers (ST). STATSports 10 Hz GNSS Apex units were used to monitor external load parameters such as distance, high-speed running (HSR), sprinting distance, accelerations, and decelerations. The linear mixed model analysis found differences between formats (*p* < 0.001) for the rate of perceived exertion (RPE), distance, HSR, sprinting, accelerations, and decelerations. Differences were found between positions for HSR (*p* = 0.004), sprinting (*p* = 0.006), and decelerations (*p* < 0.001). Moreover, a significant difference was found between sided game types (*p* < 0.001) for RPE, distance, HSR, sprinting, accelerations, and decelerations. In conclusion, some sided games formats are more suitable for specific load-specific parameters (e.g., distance per minute, HSR, and sprinting are greater during LSG). The number of accelerations and decelerations is higher in MSG compared to other formats. Finally, players' positions influenced external load metrics, specifically HSR and decelerations but not RPE and distance.

## Introduction

Football training aims to develop physical capacities, tactical and technical skills to compete during matches (1). In the latest years, sided games, which are categorized as small (SSG), medium (MSG), and large (LSG) formats, have been commonly used by coaches for simultaneously training these capacities and skills (2). Sided games are ball-based drills typically played on smaller pitch areas than regular games, with a fewer players and sometimes using modified rules to achieve specific physical, technical, and tactical aims (3, 4). From a conditioning perspective, sided games can improve aerobic and anaerobic fitness, acceleration and deceleration capacities (4, 5). Still, it has been reported that they may struggle to replicate the high-speed running (HSR) and sprinting demands of football matches (6, 7).

From a training management perspective, previous research found that sided games can be manipulated by changing the pitch sizes on which they are played to obtain a different relative area per player (3, 6). Indeed, HSR increases when larger relative pitch areas are used, while more accelerations and decelerations are performed when smaller relative pitch areas are used (7–10). During a microcycle (1 week) in football, specific sided games are selected and allocated to specific days (e.g., following the principles of tactical periodization) to achieve the desired physical stimuli to the players (11, 12). Sided games are commonly manipulated by changing the number of players involved in the sided games. For instance, SSG format includes 2vs2, 3vs3, and 4vs4, MSG format includes 5vs5, 6vs6, and 7vs7, and LSG formats included 8vs8, 9vs9, and 10vs10 sided games (7). Although a large body of research has investigated different aspects of sided games, we know that acceleration and deceleration demands as well as HSR and sprinting distances are highly variable among studies (7, 10). However, minimal information about thorough analysis from SSG (i.e., 2vs2) to LSG (i.e., 10vs10) within the same football team is available. This could be particularly interesting because it is known that the “one size fits all” approach to sided games actually does not work (13). Furthermore, it is known that players' positions play a role in the external load demands during matches (14). External positions usually require more HSR distance than center defenders or attackers, or in other cases, central positions require a greater number of accelerations and decelerations than external positions (14, 15). This could also be the case for external load demands during sided games, but it is unclear which games allow for different physical demands between positions. Coaches interested in manipulating training demands among positions during their training microcycle should understand these differences.

The quantification of training load is usually categorized into internal and external training load (16, 17). Internal training load is frequently assessed by collecting a player's rating of perceived exertion (RPE) (18, 19). RPE is cheap, easily administered and can capture a player's overall perceived load at the end of drills and sessions (20). Moreover, previous research reported that RPE score is correlated with other physiological measures such as heart rate and blood lactate (18). External training load is commonly monitored using the global navigation satellite system (GNSS) (2, 21, 22). GNSS has been proven to be valid and reliable to assess distance during linear and sport-specific tasks and determining peak speed (21, 23). Moreover, such technology can quantify the number of accelerations and decelerations during drills and sessions, which are critical parameters to consider during the weekly training plan in football (10). Because the difference in internal and external training load between sided games formats as well as the effect of players' positions on such parameters are not clear, a specific study investigating these variables is needed. Therefore, we aimed to verify if internal and external load parameters were different between sided-game formats (SSG, MSG, LSG) and if players' positions influenced these parameters. Finally, we intended to clarify whether internal and external load parameters differed among sided-game types (from 2vs2 to 10vs10) in professional male football players.

## Methods

### Participants

Twenty-five male professional football players of the same club were enrolled in this study (age = 27 ± 9 years and body mass = 78 ± 14 kg) during the 2022–23 season. The inclusion criteria included the absence of illness and injuries and regular football training and competition participation. Goalkeepers (GKs) were excluded from this study, and only outfield players' match data were evaluated. The sample size estimation was calculated using G*power (Düsseldorf, Germany) for a one-way ANOVA fixed effect that indicated a total of 159 individual data points would be required to detect a *small* effect (*f *= 0.25) with 80% power and an alpha of 5%. The actual sample size of this study was 780 individual data points, with a real power of >95%, which reduced the likelihood of type 2 errors (false negative) (24). The Ethics Committee of the University of Suffolk (Ipswich, UK) approved this study (project code: RETHS22/016). Informed consent to take part in this research was signed by the club. The external training load data was recorded as part of the regular monitoring routine of the club and was only analyzed *a posteriori*. All procedures were conducted according to the Declaration of Helsinki for human studies.

### Experimental design

Sided games were categorized in formats such as SSG (*n* = 145), MSG (*n* = 431), and LSG (*n* = 204). SSG included 2vs2, 3vs3, and 4vs4 sided games; MSG included 5vs5, 6vs6, and 7vs7 sided games; LSG included 8vs8 and 10vs10 sided games (7). During these sided games goals were included as well as GKs, football balls around the pitch were available to be used when one ball was kicked off the pitch, and coaches encouraged players to increase intensity. Only players that completed the drill were included in this analysis. Players were divided into positions such as center backs (CB), fullbacks (FB), center midfielders (CM), attacking midfielders (AM), and strikers (ST). The specific number of data points per position is reported in the Supplementary Material.

#### Sided game type

(a) 2vs2 SSG played with the same rules of a match in restricted spaces, using a medium pitch format, with a relative pitch area of 121 m^2^.
(b) 3vs3 SSG played using a small pitch format, with a relative pitch area of 72 m^2^.
(c) 4vs4 SSG played using small and medium pitch formats, with a relative pitch area of 68 m^2^ and 104 m^2^, respectively.
(d) 5vs5 MSG played using small and medium pitch formats, with a relative pitch area of 72 m^2^ and 115 m^2^, respectively.
(e) 6vs6 MSG played using small and medium pitch formats, with a relative pitch area of 67 m^2^ and 140 m^2^, respectively.
(f) 7vs7 MSG played using medium pitch formats, with a relative pitch area of 102 m^2^ and 144 m^2^, respectively.
(g) 8vs8 LSG played using a medium pitch format, with a relative pitch area of 157.5 m^2^.
(h) 10vs10 LSG played using large and regular pitch formats, with a relative pitch area of 229 m^2^ and 353.4 m^2^ (108 m × 72 m), respectively.

The specific number of data points per sided game type is reported in the Supplementary Material.

SSG, MSG, and LSG formats were arbitrarily categorized as small pitch size (<99 m^2^), medium pitch size (from 100 to 199 m^2^), large pitch size (from 200 to 289 m^2^), and regular pitch size >290 m^2^, which is the minimum standard size (100 m × 64 m) of a football pitch for a professional 11-a-side game set by Fédération Internationale de Football Association. In this article, the regular pitch size used was 108 m × 72 m, equivalent to 353.4 m^2^ per player (including GKs).

#### Global navigation satellite system (GNSS)

In this study, STATSports 10 Hz GNSS Apex units (Northern Ireland, UK) were used to monitor SSG, MSG, and LSG. GNSS technology tracks multiple satellite systems (i.e., global positioning systems, GLONASS) to provide highly accurate and reliable positional information (21). Moreover, Apex units are integrated with a 100 Hz triaxial accelerometer (25). Before each training session (e.g., 15 min), the GNSS Apex units were turned on to allow the units to track an adequate number of satellites. These units reported the number of satellites tracked that ranged between 17 and 23, which is in line with previous literature (26). All data recorded by the Apex units were downloaded and elaborated by STATSports software (Apex version Sonra v4.4.17) before being exported as a CSV file for further analysis.

Previous research reported the validity and reliability of this technology during linear and soccer-specific tasks reporting an error of <2.5% (21). The reliability (inter-unit) during sprinting actions (range: 5–30 m) was *excellent* (intra-class correlation coefficient = 0.99), with a typical error of measurement of 1.85% (26).

#### External and internal load variables

Players' internal load was expressed in arbitrary units (AU) and monitored using a previously validated scale, specifically Borg's CR10. This scale assesses players' rate of perceived exertion (RPE) (18, 27). Each player gave their RPE score after the end of each sided game (28). External load metrics were quantified and reported as frequency per minute to account for the difference in time exposure. In this study, GNSS recorded metrics were distance covered (m·min^−1^), HSR distance (>19.8 km·h^−1^), and sprinting distance (>25.2 km·h^−1^) (8). The number of high-intensity accelerations (>3 m·s^−2^), and decelerations (<−3 m·s^−2^) were quantified using GNSS technology (10).

### Statistical analyses

Descriptive statistics are reported as mean ± standard deviation (SD). A Shapiro-Wilk test was used to check the assumption that the data conforms to a normal distribution and that the residuals were found normally distributed for the linear mixed model (LMM). The primary analysis was an LMM, which used the Satterthwaite method (degrees of freedom estimation based on analytical results) to assess if significant differences exist between formats (LSG, MSG and SSG; fixed effects) and players' positions (fixed effects) across several dependent variables (29). Players were considered as random effect grouping factors. During the secondary analysis, individual sided games (from 2vs2 to 10vs10, fixed effects) and players (random effects) were analyzed using again a LMM. When significant differences were found in the LMM model, an estimation of marginal means (contrasts) was performed using Holm's corrections for multiple comparisons. Estimates of 95% confidence intervals (CIs) were calculated and reported in the figures (Box Plots). Effect sizes were calculated from the *t* and *df* of the contrast and interpreted using Cohen's *d* principle as follows *trivial *< 0.2, *small* 0.2–0.6, *moderate* 0.6–1.2, *large* 1.2–2.0, *very large* > 2.0 (30). Unless otherwise stated significance was set at p < 0.05 for all tests. Statistical analyses were performed in JASP (JASP Version 0.16.13. Amsterdam, Netherlands).

## Results

**Figure 1:**
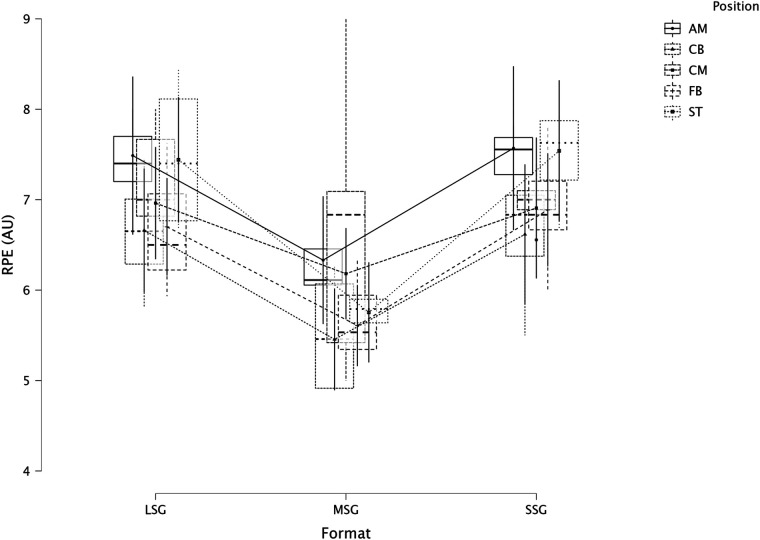
Comparison of RPE between formats and positions.

**Figure 2:**
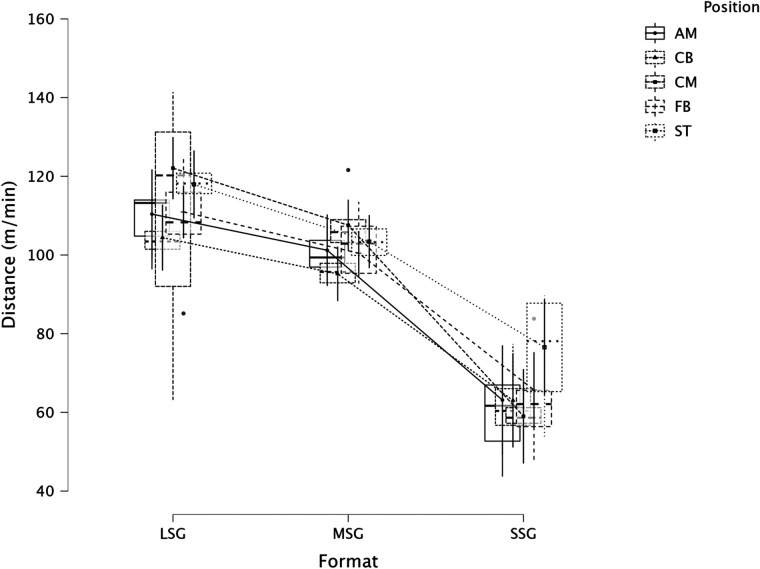
Comparison of distance between formats and positions.

**Figure 3:**
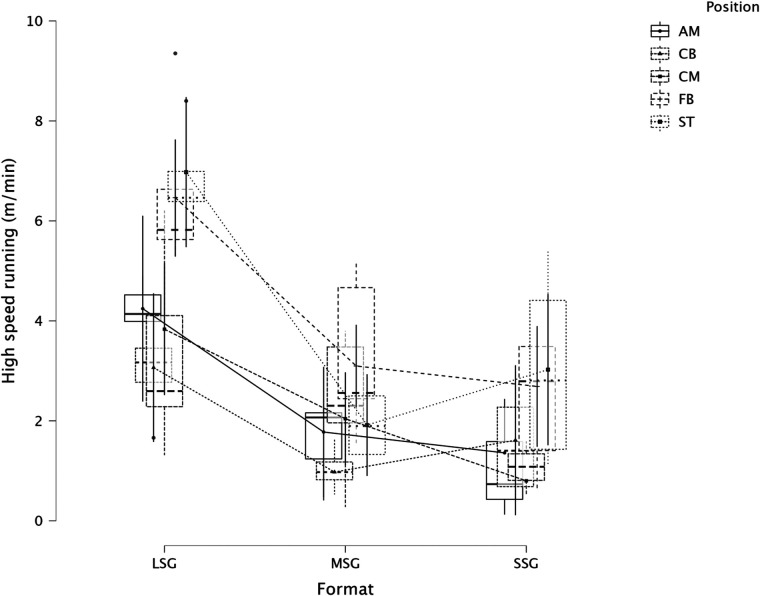
Comparison of HSR between formats and positions.

**Figure 4:**
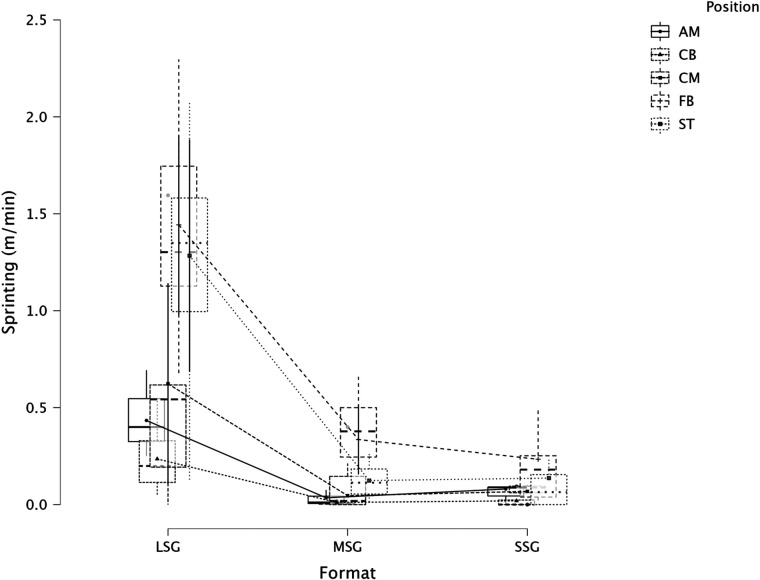
Comparison of sprinting between formats and positions.

**Figure 5:**
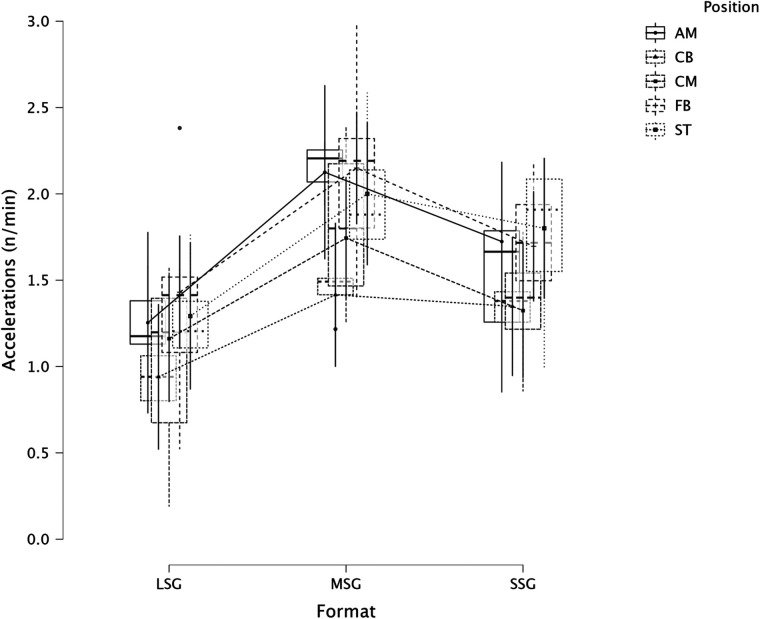
Comparison of accelerations between formats and positions.

**Figure 6:**
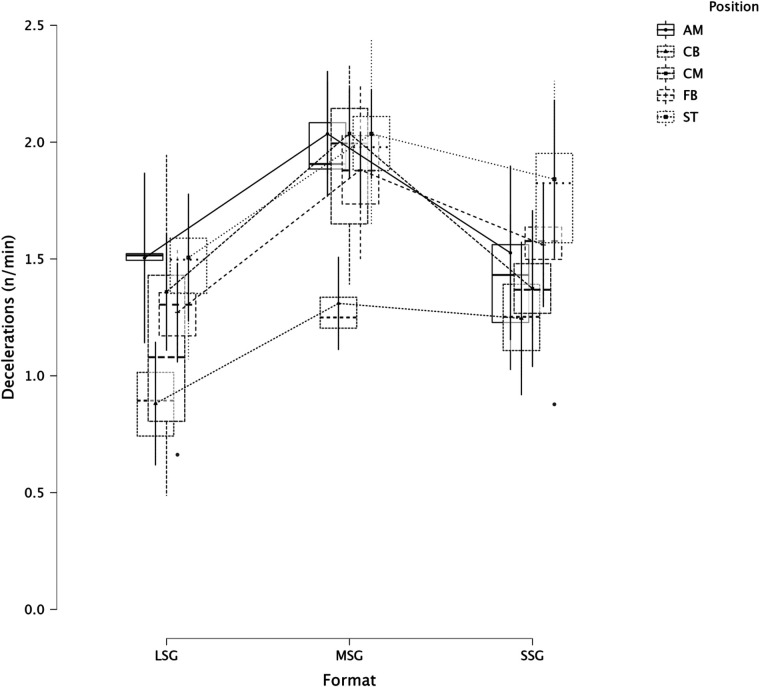
Comparison of decelerations between formats and positions.

The summary of the comparison between formats (LSG, MSG, and SSG) and positions using a LMM across several dependent variables is reported in Figure 1 (RPE), Figure 2 (distance), Figure 3 (HSR), Figure 4 (sprinting), Figure 5 (accelerations), Figure 6 (decelerations).

LMM analysis for formats (LSG, MSG, and SSG) and positions reported a significant difference between formats (*F* = 34.3, *p* < 0.001) but not for positions (*p* = 0.084) for RPE. LMM analysis reported a significant difference between formats (*F* = 167.3, *p* < 0.001) but not for positions (*p* = 0.119) for distance. LMM analysis reported a significant difference between formats (*F* = 66.1, *p* < 0.001) and positions (*p* = 0.004) for HSR. LMM analysis reported a significant difference between formats (*F* = 16.4, *p* < 0.001) and positions (*p* = 0.006) for sprinting distance. LMM analysis reported a significant difference between formats (*F* = 47.6, *p* < 0.001) but not for positions (*p* = 0.115) for accelerations. LMM analysis reported a significant difference between formats (*F* = 28.9, *p* < 0.001) and for positions (*p* < 0.001) for decelerations.

**Figure 7:**
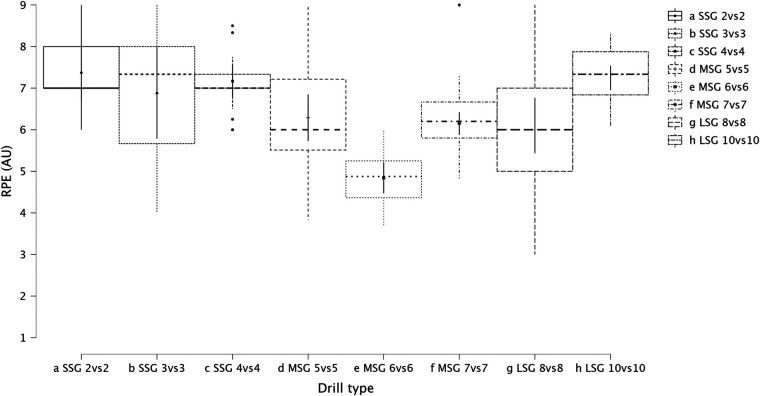
Comparison of RPE between drills type (from 2vs2 to 10vs10).

**Figure 8:**
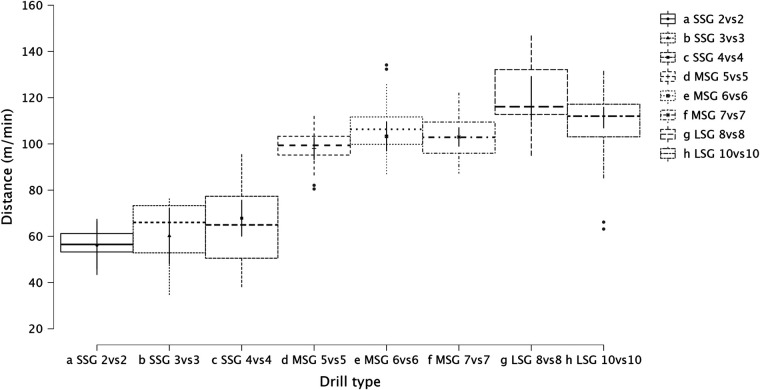
Comparison of distance between drills type (from 2vs2 to 10vs10).

**Figure 9:**
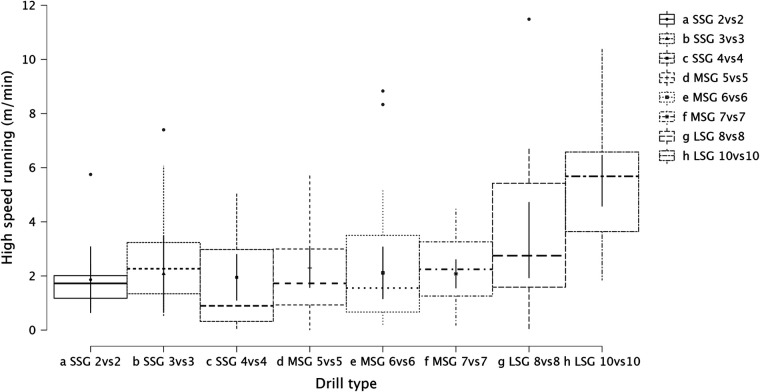
Comparison of HSR distance between drills type (from 2vs2 to 10vs10).

**Figure 10:**
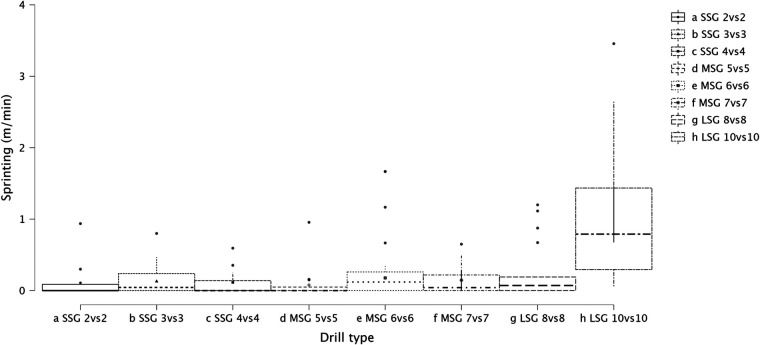
Comparison of sprinting distance between drills type (from 2vs2 to 10vs10).

**Figure 11:**
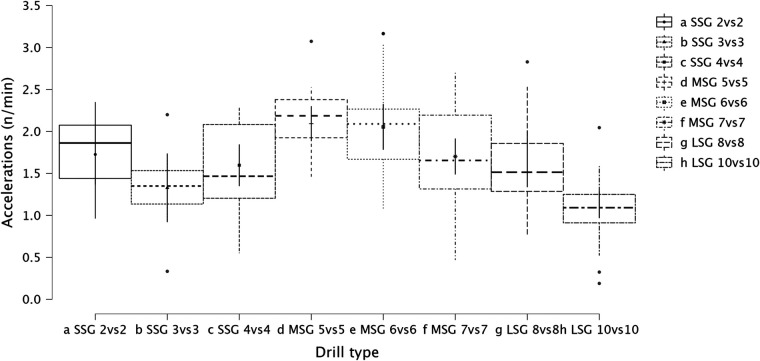
Comparison of acceleration number between drills type (from 2vs2 to 10vs10).

**Figure 12:**
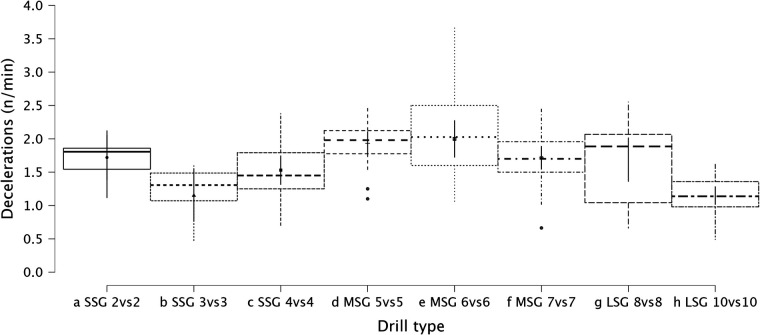
Comparison of deceleration number between drills type (from 2vs2 to 10vs10).

Summary of the secondary analysis, where individual sided games (from 2vs2 to 10vs10) were analyzed as presented in Figure 7 (RPE), Figure 8 (distance), Figure 9 (HSR), Figure 10 (sprinting), Figure 11 (accelerations), Figure 12 (decelerations). LMM analysis reported a significant difference between sided game types for RPE (*F* = 28.1, *p* < 0.001), distance (*F* = 50.6, *p* < 0.001), HSR (*F* = 14.5, *p* < 0.001), sprinting (*F* = 4.38, *p* < 0.001), accelerations (*F* = 19.8, *p* < 0.001), and decelerations (*F* = 14.8, *p* < 0.001).

The descriptive analysis of sided games formats (LSG, MSG, and SSG), players' positions (CB, FB, CM, AM, and ST), and sided game types (from 2vs2 to 10vs10) is reported in the Supplementary Material.

Estimated marginal means and 95% CIs for sided games formats (LSG, MSG, and SSG), players’ positions (CB, FB, CM, AM, and ST), and sided game types (from 2vs2 to 10vs10) were reported in the Supplementary Material.

## Discussion

This study aimed to verify, first, if internal and external load parameters were different between sided-game formats (SSG, MSG, LSG), second, if players' positions influenced these parameters, and finally, if internal and external load parameters were different among sided-game types (from 2vs2 to 10vs10) in professional male football players. We found that internal training load (RPE) changes among sided-game formats. For instance, MSG reported a lower score compared to LSG. External training load parameters are significantly different among formats where, for example, distance per minute is greater during LSG than SSG, and the number of accelerations was greater in MSG than LSG. Players' positions do not affect internal training load among the formats, while they influence some external load parameters; for example, HSR and sprinting distance are greater for ST and FB compared to CB. Finally, both internal and external load parameters were found to be different among sided games (from 2vs2 to 10vs10), where LSG 8vs8 was found to be the most demanding drill for distance covered per minute, and LSG 10 vs. 10 was found to be the most demanding drill for HSR and sprinting. On the other hand, acceleration and deceleration demands were greater in MSG 5vs5 and MSG 6vs6 compared to other formats.

### Internal and external load parameters between sided game formats

RPE was found to be significantly (*p* < 0.001) different between formats (Figure 1). Specifically, RPE in LSG was greater than RPE in MSG (*p* < 0.001, d = *very large*) while RPE in SSG was greater (*p* < 0.001, *very large*) than in MSG. From a practical perspective, practitioners can use RPE as a cheap monitoring tool for evaluating players' perceived load during sided games (18). However, when RPE is not associated with other external load data, interpreting why these differences between sided games formats exist is quite difficult. In this context, practitioners cannot understand if RPE differences among formats is due, for example, to a greater distance covered or because of a higher number of accelerations performed by the players. Therefore, we suggest practitioners use both internal and external load parameters to have a clearer picture of the demands of their sided game drills (31). Last but not least, RPE is not a pure measure of intensity because it is affected by the duration (of the drill), therefore, practitioners should be conscious of this when they compare drills of different duration. This study analyzed distance covered per minute, one of the most common parameters monitored in football (Figure 2). LSG formats obtained greater distance covered (*moderate* to *very large*) than the distance covered during MSG and SSG. While MSG (101.3 m·min^−1^) reported a significantly greater distance compared to SSG (65.6 m·min^−1^). Practitioners should preferentially use LSG and MSG when they want to replicate intensities (distance per minute) near match intensity, while SSG are clearly too small to allow for match-specific demands (7, 32). When HSR distance is analyzed, LSG reported a greater distance (5.0 m·min^−1^, *p* < 0.001) compared to MSG (2.1 m·min^−1^) and SSG (2.0 m·min^−1^). This result is in line with previous research that reported that HSR distance is greater in LSG compared to smaller formats (2, 7). Moreover, previous research reported that LSG are generally suitable to achieve sprinting speed, while smaller formats struggle to do so (8). This is supported by the data of this study, where sprinting distance is exclusively found in LSG (0.9 m·min^−1^), while MSG and SSG reported distances close to zero (around 0.1 m·min^−1^). These data confirm that, first, LSG using large spaces (>200 m^2^ per player) or with regular dimensions (>290 m^2^) are needed to achieve both HSR and sprinting distances, and second, that players very likely need to perform some running based exercises (e.g., linear sprinting activities) to actually cover an adequate amount of sprinting distance during their microcycle if SSG and MSG are mainly used (8, 33). Practitioners should also consider that matches or their “replication” using regular pitch areas (like in this study, 10vs10 = 353.4 m^2^) in training can be a potent stimulus for physical development (17, 34). Sided games are also used to generate mechanical and physiological loads in the lower limbs (35, 36); since direct quantification is highly complicated in a football context, practitioners usually quantify accelerations and decelerations (10, 37) using GNSS technology (23, 38). This study found that the number of accelerations is greater in MSG compared to LSG (*large*) and SSG (*small*). It is shown that SSG is not the best format for loading (when accelerations are the reference) players, but MSG is. Very similar results were found when decelerations were analyzed; MSG reported a significant (*p* < 0.001) greater number of decelerations compared with both LSG (*large*) and SSG (*moderate*).

### Players' positions and internal and external load parameters

In this study, we also analyzed players' positions' effect on internal and external load parameters. RPE and distance covered per minute were not significantly affected by positions, *p* = 0.084 and *p* = 0.119, respectively. Therefore, players of different positions can achieve a similar RPE or relative intensity during sided game formats. However, this was not the case when HSR distance was analyzed (Figure 3), specifically, CB (1.9 m·min^−1^) reported a significantly lower value compared to FB (4.1 m·min^−1^, *p* = 0.006) and ST (4.0 m·min^−1^, *p* = 0.014), as well as ST covered significantly more HSR than CM (2.2 m·min^−1^, *p* = 0.016). This means that coaches can use sided games to stimulate players based on their position. When sprinting distance was analyzed we found a significant difference between positions (*p* = 0.006, Figure 4), specifically, FB (0.7 m·min^−1^) outperformed the other positions such as AM (*p* = 0.025), CB (*p* = 0.005), and CM (*p* = 0.025) but they had a similar sprinting distance compared to ST (0.5 m·min^−1^, *p* = 0.329). These results highlight that while positions do not affect RPE scores or the distance per minute, they affect HSR and sprinting distance, therefore, coaches and sport scientists should consider this when they are designing their sided game drills during the training microcycle (16). Instead, accelerations were not found to be different among positions (Figure 5, *p* = 0.115), which means that all players, independently from their role, have similar mechanical demands. However, this was not the case for the number of decelerations recorded (Figure 6), since ST reported the highest number of events (1.8 n·min^−1^) compared to the other roles such as CB (1.5 n·min^−1^, *p* < 0.001) and FB (1.6 n·min^−1^, *p* = 0.026). Although these results are interesting and show that sided games' deceleration demands are affected by positions, practitioners should be quite careful because the differences are quite small (see Figure 6). Future research should evaluate if different sided game formats chronically improve some specific physical parameters more than others and if players of different positions actually improve differently.

### Internal and external load parameters among sided game types (from 2vs2 to 10vs10)

The secondary analysis of this paper assessed the internal and external load parameters among the sided game types. We found that RPE score was higher in SSG 2vs2 (RPE = 7.4) compared to MSG 5vs5 (RPE = 6.3, *p* = 0.003), MSG 6vs6 (RPE = 4.8, *p* < 0.001) and MSG 7vs7 (RPE = 6.1, *p* < 0.001) but not compared to LSG 10vs10 (RPE = 7.3, *p* = 0.636). Therefore, coaches should select drills that are very small or very large if they want to increase their players' perceived exertion (Figure 7). However, practitioners should understand that the external load parameters among these drills (SSG 2vs2 and LSG 10vs10) are very different. Therefore, the consequent mechanical and physiological adaptations will also be different although they have similar RPE values. As reported in Figure 8, LSG 10vs10 (111.4 m·min^−1^) reported the greater distance per minute among sided game types and in particular they have nearly twice the value compared to SSG 2vs2 (56.1 m·min^−1^). Previous literature reported that professional players of a similar level (English Football League One) covered a distance per minute of 106 m·min^−1^ during official matches (39). Therefore, all drills above 100 m·min^−1^ reported in Figure 8 (i.e., MSG 6vs6, MSG 7vs7, LSG 8vs8 and LSG 10vs10) would be suitable to replicate the demands of the match for these players (English League One level). However, the intensity per minute recorded in other leagues is higher than what is reported here, so practitioners should verify that their drills obtain the desired intensity (40, 41). Regarding HSR, LSG10vs10 and LSG 8vs8 (Figure 9) are the most demanding drills, for instance they had a mean intensity of 5.5 m·min^−1^ and 3.3 m·min^−1^, respectively, which is *largely* different (*p* < 0.001) compared to any SSG formats. Moreover, when the sprinting distance was assessed, LSG10vs10 were the only format which actually reported an intensity greater than 1 m·min^−1^ (Figure 10). Therefore, LSG10vs10 is the only format that is recommended to be used when coaches want to develop sprinting with their players. However, we should be aware that the overall sprinting dose is limited and may not be sufficient to achieve the aim (2, 17, 33, 42, 43). Contrariwise, regarding accelerations and decelerations, the most suitable drills for training purposes are MSG 5vs5 and MSG 6vs6, respectively (Figures 11, 12). Overall, MSG formats (i.e., 5vs5 and 6vs6) seem to offer a valid acceleration and deceleration frequencies >2 n · min^−1^. Therefore, coaches and sports scientists could use these sided games to stimulate acceleration and deceleration training doses in their players (10).

### Limitations and future directions

This study has some limitations, first, the sample enrolled in this study is a professional team in the English League One; therefore, higher or lower-level players could present different internal and external load demands compared to the ones reported here as well as coaches of other clubs could differently influence these drills with their encouragement. Second, only male players were assessed in this study; therefore, these results should be replicated with female football players to verify that what is reported here can be extended to female populations. Moreover, recent research reported that the use of individualized players' speed thresholds (e.g., sprinting speed or maximal aerobic speed) could be helpful in training load monitoring. An individualization based on the peak speed (e.g., recorded by GNSS) was not performed in this study, therefore, future research could investigate if this approach can offer additional insights (2, 17, 44). Lastly, this study did not consider any metabolic load parameter (e.g., metabolic power) or heart rate (45, 46). Future studies could verify whether these parameters differ among-sided game formats (SSG, MSG, and LSG) or if players' positions influence them.

## Conclusions

This study found that internal (i.e., RPE) and external load parameters (e.g., accelerations and sprinting distance) were different between sided-game formats (SSG, MSG, LSG) in professional football players. Some formats were more suitable to load some specific parameters. For instance, distance per minute was greater during LSG than SSG and HSR, and sprinting distance was greater in LSG compared to SSG. This study found that the number of accelerations and decelerations was higher in MSG compared to SSG and LSG, which could have interesting practical applications for coaches. Moreover, this study found that external load metrics (e.g., HSR and decelerations) were subjective to players' positions. For example, HSR and sprinting distance were greater for ST and FB than CB. However, RPE and distance per minute were not affected by positions. Coaches should be aware of the internal load and external load demands of different game formats (LSG, MSG, and SSG), as well as if players' positions can influence these load parameters that would be critical for training load planning. Finally, this study analyzed the internal and external load parameters among sided-game types (from 2vs2 to 10vs10) and found that LSG 8vs8 was the most demanding drill for distance covered per minute, LSG 10 vs. 10 was the most demanding drill for HSR and sprinting. On the other hand, acceleration and deceleration demands were greater in MSG 5vs5 and MSG 6vs6 compared to other formats. Coaches and sports scientists should consider these findings and select the most appropriate sided-game types during their training.

## Data Availability

The raw data supporting the conclusions of this article will be made available by the authors, without undue reservation.
