# Supplementary material for: Training load comparison between small, medium, and large-sided games in professional football
Source: Front Sports Act Living. 2023 May 5;5:1165242. doi: 10.3389/fspor.2023.1165242 (PMC10196496; doi:10.3389/fspor.2023.1165242)
Supplement: Supplementary file 1 [file Table1.docx]

**Supplementary material**

Table 1. Descriptive values sided game formats.

| **Descriptive Statistics** | | | | | | | | | | | | | | | | | | | | | | | | | | | | | | | | | | | | | | | | | | | | | | | | |
| --- | --- | --- | --- | --- | --- | --- | --- | --- | --- | --- | --- | --- | --- | --- | --- | --- | --- | --- | --- | --- | --- | --- | --- | --- | --- | --- | --- | --- | --- | --- | --- | --- | --- | --- | --- | --- | --- | --- | --- | --- | --- | --- | --- | --- | --- | --- | --- | --- |
|  | | **RPE** | | | | | | **Distance** | | | | | | | | **High speed running** | | | | | | | | | **Sprinting** | | | | | | | | **Accelerations** | | | | | | | | **Decelerations** | | | | | | | |
|  | | **LSG** | | **MSG** | | **SSG** | | **LSG** | | **MSG** | | | **SSG** | | | **LSG** | | | **MSG** | | | **SSG** | | | **LSG** | | | **MSG** | | | **SSG** | | **LSG** | | | **MSG** | | | **SSG** | | **LSG** | | | **MSG** | | | **SSG** | |
| Valid |  | 204 |  | 431 |  | 145 |  | 204 |  | 431 |  | 145 | |  | 204 | |  | 431 | |  | 145 | |  | 204 | |  | 431 | |  | 145 | |  | 204 |  | 431 | |  | 145 | |  | 204 |  | 431 | |  | 145 | |  |
| Missing |  | 0 |  | 0 |  | 0 |  | 0 |  | 0 |  | 0 | |  | 0 | |  | 0 | |  | 0 | |  | 0 | |  | 0 | |  | 0 | |  | 0 |  | 0 | |  | 0 | |  | 0 |  | 0 | |  | 0 | |  |
| Mean |  | 6.931 |  | 5.722 |  | 7.124 |  | 113.297 |  | 101.222 |  | 66.061 | |  | 5.246 | |  | 2.054 | |  | 2.181 | |  | 0.946 | |  | 0.140 | |  | 0.130 | |  | 1.283 |  | 1.945 | |  | 1.649 | |  | 1.282 |  | 1.836 | |  | 1.527 | |  |
| Std. Deviation |  | 1.206 |  | 1.614 |  | 1.224 |  | 19.988 |  | 24.391 |  | 22.648 | |  | 3.591 | |  | 2.508 | |  | 2.846 | |  | 1.627 | |  | 0.504 | |  | 0.358 | |  | 0.645 |  | 0.935 | |  | 0.779 | |  | 0.588 |  | 0.889 | |  | 0.672 | |  |
| Minimum |  | 3.000 |  | 0.000 |  | 3.000 |  | 57.622 |  | 0.000 |  | 5.500 | |  | 0.000 | |  | 0.000 | |  | 0.000 | |  | 0.000 | |  | 0.000 | |  | 0.000 | |  | 0.135 |  | 0.000 | |  | 0.000 | |  | 0.100 |  | 0.000 | |  | 0.000 | |  |
| Maximum |  | 9.000 |  | 9.000 |  | 9.000 |  | 154.714 |  | 165.500 |  | 127.000 | |  | 18.800 | |  | 15.500 | |  | 13.667 | |  | 10.000 | |  | 5.000 | |  | 2.000 | |  | 4.200 |  | 4.667 | |  | 5.500 | |  | 2.714 |  | 5.000 | |  | 4.000 | |  |
|  | | | | | | | | | | | | | | | | | | | | | | | | | | | | | | | | | | | | | | | | | | | | | | | | |

Large-sided games (LSG), Medium-sided games (MSG), Small-sided games (SSG), Rating perceived exertion (RPE)

Table 2a. Descriptive values for positions (RPE, Distance, High-speed Running)

| **Descriptive Statistics** | | | | | | | | | | | | | | | | | | | | | | | | | | | | | | | | | | | | | | | |
| --- | --- | --- | --- | --- | --- | --- | --- | --- | --- | --- | --- | --- | --- | --- | --- | --- | --- | --- | --- | --- | --- | --- | --- | --- | --- | --- | --- | --- | --- | --- | --- | --- | --- | --- | --- | --- | --- | --- | --- |
|  | | **RPE** | | | | | | | | | | **Distance** | | | | | | | | | | | | | | **High speed running** | | | | | | | | | | | | | |
|  | | **AM** | | **CB** | | **CM** | | **FB** | | **ST** | | **AM** | | | **CB** | | | **CM** | | | **FB** | | | **ST** | | **AM** | | | **CB** | | | **CM** | | | **FB** | | | **ST** | |
| Valid |  | 80 |  | 152 |  | 145 |  | 243 |  | 160 |  | 80 |  | 152 | |  | 145 | |  | 243 | |  | 160 | |  | 80 |  | 152 | |  | 145 | |  | 243 | |  | 160 | |  |
| Missing |  | 0 |  | 0 |  | 0 |  | 0 |  | 0 |  | 0 |  | 0 | |  | 0 | |  | 0 | |  | 0 | |  | 0 |  | 0 | |  | 0 | |  | 0 | |  | 0 | |  |
| Mean |  | 6.975 |  | 5.875 |  | 6.455 |  | 6.111 |  | 6.506 |  | 93.901 |  | 92.036 | |  | 104.233 | |  | 96.118 | |  | 102.164 | |  | 2.362 |  | 1.551 | |  | 2.555 | |  | 3.843 | |  | 3.390 | |  |
| Std. Deviation |  | 1.387 |  | 1.743 |  | 1.269 |  | 1.494 |  | 1.755 |  | 28.926 |  | 22.686 | |  | 31.603 | |  | 28.400 | |  | 26.546 | |  | 2.590 |  | 2.056 | |  | 2.763 | |  | 3.566 | |  | 3.617 | |  |
| Minimum |  | 4.000 |  | 2.000 |  | 3.000 |  | 0.000 |  | 1.000 |  | 6.000 |  | 10.500 | |  | 10.000 | |  | 1.000 | |  | 0.000 | |  | 0.000 |  | 0.000 | |  | 0.000 | |  | 0.000 | |  | 0.000 | |  |
| Maximum |  | 9.000 |  | 9.000 |  | 9.000 |  | 9.000 |  | 9.000 |  | 152.500 |  | 155.000 | |  | 165.500 | |  | 159.500 | |  | 159.500 | |  | 11.000 |  | 12.000 | |  | 13.000 | |  | 15.946 | |  | 18.800 | |  |
|  | | | | | | | | | | | | | | | | | | | | | | | | | | | | | | | | | | | | | | | |

Rating perceived exertion (RPE)

Table 2b. Descriptive values for positions (Sprinting, Accelerations, Decelerations)

| **Descriptive Statistics** | | | | | | | | | | | | | | | | | | | | | | | | | | | | | | | | | | | | | | | | | |
| --- | --- | --- | --- | --- | --- | --- | --- | --- | --- | --- | --- | --- | --- | --- | --- | --- | --- | --- | --- | --- | --- | --- | --- | --- | --- | --- | --- | --- | --- | --- | --- | --- | --- | --- | --- | --- | --- | --- | --- | --- | --- |
|  | | **Sprinting** | | | | | | | | | | | **Accelerations** | | | | | | | | | | | | | | | **Decelerations** | | | | | | | | | | | | | |
|  | | **AM** | | **CB** | | **CM** | | **FB** | | **ST** | | | **AM** | | | **CB** | | | **CM** | | | **FB** | | | **ST** | | | **AM** | | | **CB** | | | **CM** | | | **FB** | | | **ST** | |
| Valid |  | 80 |  | 152 |  | 145 |  | 243 |  | 160 |  | 80 | |  | 152 | |  | 145 | |  | 243 | |  | 160 | |  | 80 | |  | 152 | |  | 145 | |  | 243 | |  | 160 | |  |
| Missing |  | 0 |  | 0 |  | 0 |  | 0 |  | 0 |  | 0 | |  | 0 | |  | 0 | |  | 0 | |  | 0 | |  | 0 | |  | 0 | |  | 0 | |  | 0 | |  | 0 | |  |
| Mean |  | 0.134 |  | 0.059 |  | 0.253 |  | 0.590 |  | 0.455 |  | 1.780 | |  | 1.280 | |  | 1.569 | |  | 1.982 | |  | 1.831 | |  | 1.770 | |  | 1.193 | |  | 1.726 | |  | 1.653 | |  | 1.872 | |  |
| Std. Deviation |  | 0.362 |  | 0.227 |  | 0.773 |  | 1.144 |  | 1.394 |  | 0.855 | |  | 0.658 | |  | 0.856 | |  | 0.917 | |  | 0.889 | |  | 0.772 | |  | 0.577 | |  | 0.929 | |  | 0.759 | |  | 0.856 | |  |
| Minimum |  | 0.000 |  | 0.000 |  | 0.000 |  | 0.000 |  | 0.000 |  | 0.000 | |  | 0.000 | |  | 0.000 | |  | 0.000 | |  | 0.000 | |  | 0.000 | |  | 0.000 | |  | 0.000 | |  | 0.000 | |  | 0.000 | |  |
| Maximum |  | 1.857 |  | 1.714 |  | 5.200 |  | 6.200 |  | 10.000 |  | 4.000 | |  | 3.000 | |  | 4.000 | |  | 4.667 | |  | 5.500 | |  | 4.500 | |  | 3.000 | |  | 5.000 | |  | 4.000 | |  | 4.667 | |  |
|  | | | | | | | | | | | | | | | | | | | | | | | | | | | | | | | | | | | | | | | | | |

central backs (CB), fullbacks (FB), central midfielders (CM), attacking midfielders (AM), and strikers (ST)

Table 3a. Descriptive values sided-game types (from 2vs2 to 10vs10)

| **Descriptive Statistics** | | | | | | | | | | | | | | | | | |
| --- | --- | --- | --- | --- | --- | --- | --- | --- | --- | --- | --- | --- | --- | --- | --- | --- | --- |
|  | | **RPE** | | | | | | | | | | | | | | | |
|  | | **a SSG 2vs2** | | **b SSG 3vs3** | | **c SSG 4vs4** | | **d MSG 5vs5** | | **e MSG 6vs6** | | **f MSG 7vs7** | | **g LSG 8vs8** | | **h LSG 10vs10** | |
| Valid |  | 28 |  | 27 |  | 90 |  | 126 |  | 114 |  | 191 |  | 46 |  | 158 |  |
| Missing |  | 0 |  | 0 |  | 0 |  | 0 |  | 0 |  | 0 |  | 0 |  | 0 |  |
| Mean |  | 7.500 |  | 6.259 |  | 7.267 |  | 6.087 |  | 4.675 |  | 6.105 |  | 5.978 |  | 7.209 |  |
| Std. Deviation |  | 0.839 |  | 2.086 |  | 0.804 |  | 1.802 |  | 1.194 |  | 1.421 |  | 1.422 |  | 0.978 |  |
| Minimum |  | 6.000 |  | 3.000 |  | 5.000 |  | 0.000 |  | 2.000 |  | 1.000 |  | 3.000 |  | 5.000 |  |
| Maximum |  | 9.000 |  | 9.000 |  | 9.000 |  | 9.000 |  | 7.000 |  | 9.000 |  | 9.000 |  | 9.000 |  |
|  | | | | | | | | | | | | | | | | | |

Rating perceived exertion (RPE)

Table 3b. Descriptive values sided-game types (from 2vs2 to 10vs10)

| **Descriptive Statistics** | | | | | | | | | | | | | | | | | |
| --- | --- | --- | --- | --- | --- | --- | --- | --- | --- | --- | --- | --- | --- | --- | --- | --- | --- |
|  | | **Distance** | | | | | | | | | | | | | | | |
|  | | **a SSG 2vs2** | | **b SSG 3vs3** | | **c SSG 4vs4** | | **d MSG 5vs5** | | **e MSG 6vs6** | | **f MSG 7vs7** | | **g LSG 8vs8** | | **h LSG 10vs10** | |
| Valid |  | 28 |  | 27 |  | 90 |  | 126 |  | 114 |  | 191 |  | 46 |  | 158 |  |
| Missing |  | 0 |  | 0 |  | 0 |  | 0 |  | 0 |  | 0 |  | 0 |  | 0 |  |
| Mean |  | 56.529 |  | 59.857 |  | 70.889 |  | 97.864 |  | 102.250 |  | 102.824 |  | 120.224 |  | 111.280 |  |
| Std. Deviation |  | 7.526 |  | 20.870 |  | 24.933 |  | 32.666 |  | 16.219 |  | 21.845 |  | 16.180 |  | 20.576 |  |
| Minimum |  | 37.400 |  | 5.500 |  | 36.200 |  | 5.667 |  | 68.600 |  | 0.000 |  | 91.200 |  | 57.622 |  |
| Maximum |  | 71.200 |  | 79.500 |  | 127.000 |  | 165.500 |  | 160.000 |  | 155.000 |  | 151.800 |  | 154.714 |  |
|  | | | | | | | | | | | | | | | | | |

Table 3c. Descriptive values sided-game types (from 2vs2 to 10vs10)

| **Descriptive Statistics** | | | | | | | | | | | | | | | | | |
| --- | --- | --- | --- | --- | --- | --- | --- | --- | --- | --- | --- | --- | --- | --- | --- | --- | --- |
|  | | **High speed running** | | | | | | | | | | | | | | | |
|  | | **a SSG 2vs2** | | **b SSG 3vs3** | | **c SSG 4vs4** | | **d MSG 5vs5** | | **e MSG 6vs6** | | **f MSG 7vs7** | | **g LSG 8vs8** | | **h LSG 10vs10** | |
| Valid |  | 28 |  | 27 |  | 90 |  | 126 |  | 114 |  | 191 |  | 46 |  | 158 |  |
| Missing |  | 0 |  | 0 |  | 0 |  | 0 |  | 0 |  | 0 |  | 0 |  | 0 |  |
| Mean |  | 1.841 |  | 2.196 |  | 2.282 |  | 2.351 |  | 1.671 |  | 2.086 |  | 3.676 |  | 5.702 |  |
| Std. Deviation |  | 1.971 |  | 2.341 |  | 3.206 |  | 2.970 |  | 2.407 |  | 2.200 |  | 3.807 |  | 3.405 |  |
| Minimum |  | 0.100 |  | 0.000 |  | 0.000 |  | 0.000 |  | 0.000 |  | 0.000 |  | 0.000 |  | 0.000 |  |
| Maximum |  | 10.000 |  | 7.400 |  | 13.667 |  | 15.500 |  | 12.333 |  | 9.200 |  | 14.800 |  | 18.800 |  |
|  | | | | | | | | | | | | | | | | | |

Table 3d. Descriptive values sided-game types (from 2vs2 to 10vs10)

| **Descriptive Statistics** | | | | | | | | | | | | | | | | | |
| --- | --- | --- | --- | --- | --- | --- | --- | --- | --- | --- | --- | --- | --- | --- | --- | --- | --- |
|  | | **Sprinting** | | | | | | | | | | | | | | | |
|  | | **a SSG 2vs2** | | **b SSG 3vs3** | | **c SSG 4vs4** | | **d MSG 5vs5** | | **e MSG 6vs6** | | **f MSG 7vs7** | | **g LSG 8vs8** | | **h LSG 10vs10** | |
| Valid |  | 28 |  | 27 |  | 90 |  | 126 |  | 114 |  | 191 |  | 46 |  | 158 |  |
| Missing |  | 0 |  | 0 |  | 0 |  | 0 |  | 0 |  | 0 |  | 0 |  | 0 |  |
| Mean |  | 0.108 |  | 0.128 |  | 0.138 |  | 0.090 |  | 0.179 |  | 0.150 |  | 0.234 |  | 1.154 |  |
| Std. Deviation |  | 0.368 |  | 0.251 |  | 0.385 |  | 0.480 |  | 0.614 |  | 0.443 |  | 0.646 |  | 1.763 |  |
| Minimum |  | 0.000 |  | 0.000 |  | 0.000 |  | 0.000 |  | 0.000 |  | 0.000 |  | 0.000 |  | 0.000 |  |
| Maximum |  | 1.875 |  | 0.800 |  | 2.000 |  | 5.000 |  | 3.333 |  | 2.800 |  | 3.200 |  | 10.000 |  |
|  | | | | | | | | | | | | | | | | | |

Table 3e. Descriptive values sided-game types (from 2vs2 to 10vs10)

| **Descriptive Statistics** | | | | | | | | | | | | | | | | | |
| --- | --- | --- | --- | --- | --- | --- | --- | --- | --- | --- | --- | --- | --- | --- | --- | --- | --- |
|  | | **Accelerations** | | | | | | | | | | | | | | | |
|  | | **a SSG 2vs2** | | **b SSG 3vs3** | | **c SSG 4vs4** | | **d MSG 5vs5** | | **e MSG 6vs6** | | **f MSG 7vs7** | | **g LSG 8vs8** | | **h LSG 10vs10** | |
| Valid |  | 28 |  | 27 |  | 90 |  | 126 |  | 114 |  | 191 |  | 46 |  | 158 |  |
| Missing |  | 0 |  | 0 |  | 0 |  | 0 |  | 0 |  | 0 |  | 0 |  | 0 |  |
| Mean |  | 1.758 |  | 1.355 |  | 1.703 |  | 2.123 |  | 2.100 |  | 1.735 |  | 1.803 |  | 1.132 |  |
| Std. Deviation |  | 0.529 |  | 0.775 |  | 0.830 |  | 0.990 |  | 0.848 |  | 0.907 |  | 0.742 |  | 0.528 |  |
| Minimum |  | 0.625 |  | 0.000 |  | 0.250 |  | 0.000 |  | 0.000 |  | 0.000 |  | 0.500 |  | 0.135 |  |
| Maximum |  | 2.600 |  | 3.000 |  | 5.500 |  | 4.500 |  | 4.333 |  | 4.667 |  | 4.200 |  | 3.125 |  |
|  | | | | | | | | | | | | | | | | | |

Table 3f. Descriptive values sided-game types (from 2vs2 to 10vs10)

| **Descriptive Statistics** | | | | | | | | | | | | | | | | | |
| --- | --- | --- | --- | --- | --- | --- | --- | --- | --- | --- | --- | --- | --- | --- | --- | --- | --- |
|  | | **Accelerations** | | | | | | | | | | | | | | | |
|  | | **a SSG 2vs2** | | **b SSG 3vs3** | | **c SSG 4vs4** | | **d MSG 5vs5** | | **e MSG 6vs6** | | **f MSG 7vs7** | | **g LSG 8vs8** | | **h LSG 10vs10** | |
| Valid |  | 28 |  | 27 |  | 90 |  | 126 |  | 114 |  | 191 |  | 46 |  | 158 |  |
| Missing |  | 0 |  | 0 |  | 0 |  | 0 |  | 0 |  | 0 |  | 0 |  | 0 |  |
| Mean |  | 1.758 |  | 1.355 |  | 1.703 |  | 2.123 |  | 2.100 |  | 1.735 |  | 1.803 |  | 1.132 |  |
| Std. Deviation |  | 0.529 |  | 0.775 |  | 0.830 |  | 0.990 |  | 0.848 |  | 0.907 |  | 0.742 |  | 0.528 |  |
| Minimum |  | 0.625 |  | 0.000 |  | 0.250 |  | 0.000 |  | 0.000 |  | 0.000 |  | 0.500 |  | 0.135 |  |
| Maximum |  | 2.600 |  | 3.000 |  | 5.500 |  | 4.500 |  | 4.333 |  | 4.667 |  | 4.200 |  | 3.125 |  |
|  | | | | | | | | | | | | | | | | | |

Table 4. Estimated marginal means and 95% CIs for sided-games formats (LSG, MSG, and SSG).

Table 4a.

| **Estimated Marginal Means (RPE)** | | | | | | | | | | | | | |
| --- | --- | --- | --- | --- | --- | --- | --- | --- | --- | --- | --- | --- | --- |
|  | | | | | | | | | | **95% CI** | | | |
| **Row** | | **Format** | | **Estimate** | | **SE** | | **df** | | **Lower** | | **Upper** | |
| 1 |  | LSG |  | 6.992 |  | 0.145 |  | 22.542 |  | 6.691 |  | 7.293 |  |
| 2 |  | MSG |  | 5.819 |  | 0.118 |  | 18.176 |  | 5.570 |  | 6.067 |  |
| 3 |  | SSG |  | 7.070 |  | 0.163 |  | 29.823 |  | 6.736 |  | 7.403 |  |
|  | | | | | | | | | | | | | |

Table 4b.

| **Estimated Marginal Means (Distance)** | | | | | | | | | | | | | |
| --- | --- | --- | --- | --- | --- | --- | --- | --- | --- | --- | --- | --- | --- |
|  | | | | | | | | | | **95% CI** | | | |
| **Row** | | **Format** | | **Estimate** | | **SE** | | **df** | | **Lower** | | **Upper** | |
| 1 |  | LSG |  | 113.276 |  | 2.179 |  | 21.638 |  | 108.752 |  | 117.799 |  |
| 2 |  | MSG |  | 101.361 |  | 1.611 |  | 23.130 |  | 98.030 |  | 104.692 |  |
| 3 |  | SSG |  | 65.611 |  | 2.521 |  | 16.622 |  | 60.283 |  | 70.939 |  |
|  | | | | | | | | | | | | | |

Table 4c.

| **Estimated Marginal Means (High-speed running)** | | | | | | | | | | | | | | |
| --- | --- | --- | --- | --- | --- | --- | --- | --- | --- | --- | --- | --- | --- | --- |
|  | | | | | | | | | | | **95% CI** | | | |
| **Row** | | **Format** | | **Estimate** | | **SE** | | **df** | | | **Lower** | | **Upper** | |
| 1 |  | LSG |  | 5.019 |  | 0.415 |  | 23.844 |  | 4.162 | |  | 5.876 |  |
| 2 |  | MSG |  | 2.076 |  | 0.242 |  | 20.441 |  | 1.572 | |  | 2.580 |  |
| 3 |  | SSG |  | 1.960 |  | 0.313 |  | 20.169 |  | 1.307 | |  | 2.613 |  |
|  | | | | | | | | | | | | | | |

Table 4d.

| **Estimated Marginal Means (Sprinting)** | | | | | | | | | | | | | | |
| --- | --- | --- | --- | --- | --- | --- | --- | --- | --- | --- | --- | --- | --- | --- |
|  | | | | | | | | | | | **95% CI** | | | |
| **Row** | | **Format** | | **Estimate** | | **SE** | | **df** | | | **Lower** | | **Upper** | |
| 1 |  | LSG |  | 0.877 |  | 0.148 |  | 25.166 |  | 0.574 | |  | 1.181 |  |
| 2 |  | MSG |  | 0.137 |  | 0.048 |  | 47.591 |  | 0.042 | |  | 0.233 |  |
| 3 |  | SSG |  | 0.121 |  | 0.072 |  | 245.121 |  | -0.021 | |  | 0.263 |  |
|  | | | | | | | | | | | | | | |

Table 4e.

| **Estimated Marginal Means (Accelerations)** | | | | | | | | | | | | | |
| --- | --- | --- | --- | --- | --- | --- | --- | --- | --- | --- | --- | --- | --- |
|  | | | | | | | | | | **95% CI** | | | |
| **Row** | | **Format** | | **Estimate** | | **SE** | | **df** | | **Lower** | | **Upper** | |
| 1 |  | LSG |  | 1.238 |  | 0.086 |  | 28.147 |  | 1.063 |  | 1.414 |  |
| 2 |  | MSG |  | 1.887 |  | 0.094 |  | 25.096 |  | 1.693 |  | 2.081 |  |
| 3 |  | SSG |  | 1.581 |  | 0.081 |  | 32.213 |  | 1.416 |  | 1.747 |  |
|  | | | | | | | | | | | | | |

Table 4f.

| **Estimated Marginal Means (Decelerations)** | | | | | | | | | | | | | |
| --- | --- | --- | --- | --- | --- | --- | --- | --- | --- | --- | --- | --- | --- |
|  | | | | | | | | | | **95% CI** | | | |
| **Row** | | **Format** | | **Estimate** | | **SE** | | **df** | | **Lower** | | **Upper** | |
| 1 |  | LSG |  | 1.265 |  | 0.069 |  | 29.693 |  | 1.125 |  | 1.405 |  |
| 2 |  | MSG |  | 1.827 |  | 0.075 |  | 23.757 |  | 1.671 |  | 1.982 |  |
| 3 |  | SSG |  | 1.507 |  | 0.073 |  | 35.646 |  | 1.359 |  | 1.656 |  |
|  | | | | | | | | | | | | | |

Table 5. Estimated marginal means and 95% CIs for players’ positions (CB, FB, CM, AM, and ST)

Table 5a.

| **Estimated Marginal Means (RPE)** | | | | | | | | | | | | | |
| --- | --- | --- | --- | --- | --- | --- | --- | --- | --- | --- | --- | --- | --- |
|  | | | | | | | | | | **95% CI** | | | |
| **Row** | | **Position** | | **Estimate** | | **SE** | | **df** | | **Lower** | | **Upper** | |
| 1 |  | AM |  | 7.129 |  | 0.269 |  | 17.399 |  | 6.563 |  | 7.696 |  |
| 2 |  | CB |  | 6.244 |  | 0.223 |  | 15.642 |  | 5.771 |  | 6.717 |  |
| 3 |  | CM |  | 6.684 |  | 0.203 |  | 21.246 |  | 6.262 |  | 7.105 |  |
| 4 |  | FB |  | 6.398 |  | 0.174 |  | 15.386 |  | 6.028 |  | 6.769 |  |
| 5 |  | ST |  | 6.912 |  | 0.221 |  | 14.919 |  | 6.440 |  | 7.383 |  |
|  | | | | | | | | | | | | | |
| Note.  Results are averaged over the levels of: Format. | | | | | | | | | | | | | |

Table 5b

| **Estimated Marginal Means (Distance)** | | | | | | | | | | | | | |
| --- | --- | --- | --- | --- | --- | --- | --- | --- | --- | --- | --- | --- | --- |
|  | | | | | | | | | | **95% CI** | | | |
| **Row** | | **Position** | | **Estimate** | | **SE** | | **df** | | **Lower** | | **Upper** | |
| 1 |  | AM |  | 2.460 |  | 0.581 |  | 19.783 |  | 1.247 |  | 3.672 |  |
| 2 |  | CB |  | 1.884 |  | 0.484 |  | 17.810 |  | 0.866 |  | 2.902 |  |
| 3 |  | CM |  | 2.225 |  | 0.433 |  | 23.774 |  | 1.331 |  | 3.119 |  |
| 4 |  | FB |  | 4.083 |  | 0.378 |  | 18.004 |  | 3.288 |  | 4.878 |  |
| 5 |  | ST |  | 3.972 |  | 0.482 |  | 17.245 |  | 2.956 |  | 4.988 |  |
|  | | | | | | | | | | | | | |
| Note.  Results are averaged over the levels of: Format. | | | | | | | | | | | | | |

Table 5c

| **Estimated Marginal Means (High-speed running)** | | | | | | | | | | | | | |
| --- | --- | --- | --- | --- | --- | --- | --- | --- | --- | --- | --- | --- | --- |
|  | | | | | | | | | | **95% CI** | | | |
| **Row** | | **Position** | | **Estimate** | | **SE** | | **df** | | **Lower** | | **Upper** | |
| 1 |  | AM |  | 2.460 |  | 0.581 |  | 19.783 |  | 1.247 |  | 3.672 |  |
| 2 |  | CB |  | 1.884 |  | 0.484 |  | 17.810 |  | 0.866 |  | 2.902 |  |
| 3 |  | CM |  | 2.225 |  | 0.433 |  | 23.774 |  | 1.331 |  | 3.119 |  |
| 4 |  | FB |  | 4.083 |  | 0.378 |  | 18.004 |  | 3.288 |  | 4.878 |  |
| 5 |  | ST |  | 3.972 |  | 0.482 |  | 17.245 |  | 2.956 |  | 4.988 |  |
|  | | | | | | | | | | | | | |
| Note.  Results are averaged over the levels of: Format. | | | | | | | | | | | | | |

Table 5d

| **Estimated Marginal Means (Sprinting)** | | | | | | | | | | | | | |
| --- | --- | --- | --- | --- | --- | --- | --- | --- | --- | --- | --- | --- | --- |
|  | | | | | | | | | | **95% CI** | | | |
| **Row** | | **Position** | | **Estimate** | | **SE** | | **df** | | **Lower** | | **Upper** | |
| 1 |  | AM |  | 0.183 |  | 0.151 |  | 23.984 |  | -0.128 |  | 0.494 |  |
| 2 |  | CB |  | 0.089 |  | 0.125 |  | 20.902 |  | -0.170 |  | 0.348 |  |
| 3 |  | CM |  | 0.246 |  | 0.114 |  | 31.055 |  | 0.014 |  | 0.478 |  |
| 4 |  | FB |  | 0.671 |  | 0.096 |  | 20.019 |  | 0.471 |  | 0.870 |  |
| 5 |  | ST |  | 0.515 |  | 0.123 |  | 19.418 |  | 0.258 |  | 0.772 |  |
|  | | | | | | | | | | | | | |
| Note.  Results are averaged over the levels of: Format. | | | | | | | | | | | | | |

Table 5e

| **Estimated Marginal Means (Accelerations)** | | | | | | | | | | | | | |
| --- | --- | --- | --- | --- | --- | --- | --- | --- | --- | --- | --- | --- | --- |
|  | | | | | | | | | | **95% CI** | | | |
| **Row** | | **Position** | | **Estimate** | | **SE** | | **df** | | **Lower** | | **Upper** | |
| 1 |  | AM |  | 1.701 |  | 0.196 |  | 19.302 |  | 1.290 |  | 2.112 |  |
| 2 |  | CB |  | 1.235 |  | 0.166 |  | 17.733 |  | 0.886 |  | 1.583 |  |
| 3 |  | CM |  | 1.410 |  | 0.142 |  | 23.315 |  | 1.115 |  | 1.704 |  |
| 4 |  | FB |  | 1.759 |  | 0.128 |  | 18.304 |  | 1.490 |  | 2.028 |  |
| 5 |  | ST |  | 1.698 |  | 0.165 |  | 17.233 |  | 1.351 |  | 2.045 |  |
|  | | | | | | | | | | | | | |
| Note.  Results are averaged over the levels of: Format. | | | | | | | | | | | | | |

Table 5f.

| **Estimated Marginal Means (Decelerations)** | | | | | | | | | | | | | |
| --- | --- | --- | --- | --- | --- | --- | --- | --- | --- | --- | --- | --- | --- |
|  | | | | | | | | | | **95% CI** | | | |
| **Row** | | **Position** | | **Estimate** | | **SE** | | **df** | | **Lower** | | **Upper** | |
| 1 |  | AM |  | 1.689 |  | 0.087 |  | 762.832 |  | 1.517 |  | 1.860 |  |
| 2 |  | CB |  | 1.145 |  | 0.070 |  | 759.175 |  | 1.008 |  | 1.283 |  |
| 3 |  | CM |  | 1.590 |  | 0.069 |  | 768.447 |  | 1.454 |  | 1.726 |  |
| 4 |  | FB |  | 1.571 |  | 0.052 |  | 764.727 |  | 1.468 |  | 1.673 |  |
| 5 |  | ST |  | 1.794 |  | 0.067 |  | 770.282 |  | 1.663 |  | 1.925 |  |
|  | | | | | | | | | | | | | |
| Note.  Results are averaged over the levels of: Format. | | | | | | | | | | | | | |

Table 6. Estimated marginal means and 95% CIs for sided-game types (from 2vs2 to 10vs10)

Table 6 a

| **Estimated Marginal Means (RPE)** | | | | | | | | | | | | | |
| --- | --- | --- | --- | --- | --- | --- | --- | --- | --- | --- | --- | --- | --- |
|  | | | | | | | | | | **95% CI** | | | |
| **Row** | | **Drill type** | | **Estimate** | | **SE** | | **df** | | **Lower** | | **Upper** | |
| 1 |  | a SSG 2vs2 |  | 7.370 |  | 0.277 |  | 28.309 |  | 6.804 |  | 7.936 |  |
| 2 |  | b SSG 3vs3 |  | 6.885 |  | 0.444 |  | 12.037 |  | 5.917 |  | 7.853 |  |
| 3 |  | c SSG 4vs4 |  | 7.167 |  | 0.170 |  | 28.540 |  | 6.819 |  | 7.515 |  |
| 4 |  | d MSG 5vs5 |  | 6.287 |  | 0.265 |  | 17.590 |  | 5.729 |  | 6.846 |  |
| 5 |  | e MSG 6vs6 |  | 4.844 |  | 0.167 |  | 16.678 |  | 4.491 |  | 5.196 |  |
| 6 |  | f MSG 7vs7 |  | 6.148 |  | 0.130 |  | 18.785 |  | 5.877 |  | 6.420 |  |
| 7 |  | g LSG 8vs8 |  | 6.103 |  | 0.294 |  | 16.195 |  | 5.480 |  | 6.726 |  |
| 8 |  | h LSG 10vs10 |  | 7.247 |  | 0.139 |  | 27.385 |  | 6.963 |  | 7.531 |  |
|  | | | | | | | | | | | | | |

Table 6b

| **Estimated Marginal Means (Distance)** | | | | | | | | | |
| --- | --- | --- | --- | --- | --- | --- | --- | --- | --- |
|  | | | | | | **95% CI** | | | |
| **Drill type** | | **Estimate** | | **SE** | | **Lower** | | **Upper** | |
| a SSG 2vs2 |  | 56.099 |  | 4.153 |  | 47.958 |  | 64.239 |  |
| b SSG 3vs3 |  | 60.186 |  | 4.318 |  | 51.723 |  | 68.648 |  |
| c SSG 4vs4 |  | 67.856 |  | 3.636 |  | 60.729 |  | 74.983 |  |
| d MSG 5vs5 |  | 97.988 |  | 2.146 |  | 93.781 |  | 102.195 |  |
| e MSG 6vs6 |  | 103.294 |  | 2.819 |  | 97.769 |  | 108.818 |  |
| f MSG 7vs7 |  | 102.999 |  | 1.959 |  | 99.161 |  | 106.838 |  |
| g LSG 8vs8 |  | 119.725 |  | 3.952 |  | 111.978 |  | 127.472 |  |
| h LSG 10vs10 |  | 111.400 |  | 2.153 |  | 107.180 |  | 115.620 |  |
|  | | | | | | | | | |

Table 6c

| **Estimated Marginal Means (High-speed running)** | | | | | | | | | | | | | |
| --- | --- | --- | --- | --- | --- | --- | --- | --- | --- | --- | --- | --- | --- |
|  | | | | | | | | | | **95% CI** | | | |
| **Row** | | **Drill type** | | **Estimate** | | **SE** | | **df** | | **Lower** | | **Upper** | |
| 1 |  | a SSG 2vs2 |  | 1.862 |  | 0.490 |  | 92.856 |  | 0.888 |  | 2.836 |  |
| 2 |  | b SSG 3vs3 |  | 2.081 |  | 0.503 |  | 63.652 |  | 1.075 |  | 3.086 |  |
| 3 |  | c SSG 4vs4 |  | 1.948 |  | 0.391 |  | 24.731 |  | 1.142 |  | 2.755 |  |
| 4 |  | d MSG 5vs5 |  | 2.294 |  | 0.337 |  | 20.368 |  | 1.592 |  | 2.996 |  |
| 5 |  | e MSG 6vs6 |  | 2.114 |  | 0.444 |  | 12.167 |  | 1.148 |  | 3.080 |  |
| 6 |  | f MSG 7vs7 |  | 2.079 |  | 0.252 |  | 26.822 |  | 1.561 |  | 2.597 |  |
| 7 |  | g LSG 8vs8 |  | 3.325 |  | 0.620 |  | 18.280 |  | 2.024 |  | 4.626 |  |
| 8 |  | h LSG 10vs10 |  | 5.519 |  | 0.455 |  | 24.501 |  | 4.581 |  | 6.457 |  |
|  | | | | | | | | | | | | | |

Table 6d

| **Estimated Marginal Means (Sprinting)** | | | | | | | | | | | | | |
| --- | --- | --- | --- | --- | --- | --- | --- | --- | --- | --- | --- | --- | --- |
|  | | | | | | | | | | **95% CI** | | | |
| **Row** | | **Drill type** | | **Estimate** | | **SE** | | **df** | | **Lower** | | **Upper** | |
| 1 |  | a SSG 2vs2 |  | 0.108 |  | 0.153 |  | 750.114 |  | -0.191 |  | 0.408 |  |
| 2 |  | b SSG 3vs3 |  | 0.128 |  | 0.155 |  | 732.067 |  | -0.177 |  | 0.434 |  |
| 3 |  | c SSG 4vs4 |  | 0.115 |  | 0.087 |  | 217.824 |  | -0.057 |  | 0.286 |  |
| 4 |  | d MSG 5vs5 |  | 0.078 |  | 0.073 |  | 304.354 |  | -0.066 |  | 0.221 |  |
| 5 |  | e MSG 6vs6 |  | 0.177 |  | 0.079 |  | 102.249 |  | 0.020 |  | 0.335 |  |
| 6 |  | f MSG 7vs7 |  | 0.146 |  | 0.066 |  | 61.876 |  | 0.015 |  | 0.277 |  |
| 7 |  | g LSG 8vs8 |  | 0.206 |  | 0.120 |  | 347.559 |  | -0.031 |  | 0.443 |  |
| 8 |  | h LSG 10vs10 |  | 1.068 |  | 0.188 |  | 24.652 |  | 0.681 |  | 1.456 |  |
|  | | | | | | | | | | | | | |

Table 6e

| **Estimated Marginal Means (Accelerations)** | | | | | | | | | | | |
| --- | --- | --- | --- | --- | --- | --- | --- | --- | --- | --- | --- |
|  | | | | | | | | **95% CI** | | | |
| **Drill type** | | **Estimate** | | **SE** | | **df** | | **Lower** | | **Upper** | |
| a SSG 2vs2 |  | 1.726 |  | 0.146 |  | 52.805 |  | 1.434 |  | 2.018 |  |
| b SSG 3vs3 |  | 1.328 |  | 0.149 |  | 45.630 |  | 1.029 |  | 1.628 |  |
| c SSG 4vs4 |  | 1.598 |  | 0.113 |  | 19.630 |  | 1.361 |  | 1.834 |  |
| d MSG 5vs5 |  | 2.093 |  | 0.096 |  | 27.614 |  | 1.896 |  | 2.290 |  |
| e MSG 6vs6 |  | 2.054 |  | 0.125 |  | 27.382 |  | 1.799 |  | 2.310 |  |
| f MSG 7vs7 |  | 1.702 |  | 0.103 |  | 25.402 |  | 1.491 |  | 1.913 |  |
| g LSG 8vs8 |  | 1.675 |  | 0.144 |  | 37.860 |  | 1.383 |  | 1.967 |  |
| h LSG 10vs10 |  | 1.127 |  | 0.075 |  | 29.593 |  | 0.973 |  | 1.280 |  |
|  | | | | | | | | | | | |

Table 6f

| **Estimated Marginal Means (Decelerations)** | | | | | | | | | |
| --- | --- | --- | --- | --- | --- | --- | --- | --- | --- |
|  | | | | | | **95% CI** | | | |
| **Drill type** | | **Estimate** | | **SE** | | **Lower** | | **Upper** | |
| a SSG 2vs2 |  | 1.720 |  | 0.134 |  | 1.457 |  | 1.983 |  |
| b SSG 3vs3 |  | 1.150 |  | 0.137 |  | 0.880 |  | 1.419 |  |
| c SSG 4vs4 |  | 1.530 |  | 0.098 |  | 1.338 |  | 1.723 |  |
| d MSG 5vs5 |  | 1.933 |  | 0.081 |  | 1.775 |  | 2.092 |  |
| e MSG 6vs6 |  | 1.999 |  | 0.128 |  | 1.747 |  | 2.250 |  |
| f MSG 7vs7 |  | 1.716 |  | 0.084 |  | 1.552 |  | 1.880 |  |
| g LSG 8vs8 |  | 1.688 |  | 0.139 |  | 1.414 |  | 1.961 |  |
| h LSG 10vs10 |  | 1.149 |  | 0.063 |  | 1.025 |  | 1.274 |  |
|  | | | | | | | | | |
